# Assessing Executive Dysfunction in Neurodegenerative Disorders: A Critical Review of Brief Neuropsychological Tools

**DOI:** 10.3389/fnagi.2017.00369

**Published:** 2017-11-09

**Authors:** Helena S. Moreira, Ana S. Costa, São L. Castro, César F. Lima, Selene G. Vicente

**Affiliations:** ^1^Faculty of Psychology and Education Sciences, Centre for Psychology, University of Porto, Porto, Portugal; ^2^Neurology Department, Hospital de Braga, Braga, Portugal; ^3^Institute of Cognitive Neuroscience, University College London, London, United Kingdom; ^4^Instituto Universitário de Lisboa (ISCTE-IUL), Lisbon, Portugal

**Keywords:** aging, cognitive impairment, executive functions, neuropsychological assessment, neurodegenerative disorders, neuropsychological screening, psychometric properties, review

## Abstract

Executive function (EF) has been defined as a multifaceted construct that involves a variety of high-level cognitive abilities such as planning, working memory, mental flexibility, and inhibition. Being able to identify deficits in EF is important for the diagnosis and monitoring of several neurodegenerative disorders, and thus their assessment is a topic of much debate. In particular, there has been a growing interest in the development of neuropsychological screening tools that can potentially provide a reliable quick measure of EF. In this review, we critically discuss the four screening tools of EF currently available in the literature: Executive Interview-25 (EXIT 25), Frontal Assessment Battery (FAB), INECO Frontal Screening (IFS), and FRONTIER Executive Screen (FES). We first describe their features, and then evaluate their psychometric properties, the existing evidence on their neural correlates, and the empirical work that has been conducted in clinical populations. We conclude that the four screening tools generally present appropriate psychometric properties, and are sensitive to impairments in EF in several neurodegenerative conditions. However, more research will be needed mostly with respect to normative data and neural correlates, and to determine the extent to which these tools add specific information to the one provided by global cognition screening tests. More research directly comparing the available tools with each other will also be important to establish in which conditions each of them can be most useful.

## Introduction

Aging is typically associated with a subtle decline in cognitive functioning, seen even in healthy individuals (Wild-Wall et al., 2011; Pertl et al., 2017). Crucially, though, aging is also a risk factor for neurodegenerative dementias, such as Alzheimer's and Parkinson's Diseases (Horton and Wedding, 2008). Cognitive screening tests are important to diagnose these conditions and to assess their evolution. Since they provide practical and cost-effective evaluations, using them can be extremely valuable for healthcare systems, that are often under pressure to reduce costs and operate under time constraints (Larner, 2013a). Guidelines for screening tests have been presented by the Committee on Research of the American Neuropsychiatric Association (Malloy et al., 1997): they should be brief (<15 min), easy to administer, and present appropriate sensitivity, specificity, test-retest, and inter-rater validities. In primary care, clinicians typically rely on global cognition screening tools such as the Addenbrooke's Cognitive Examination (ACE; Mathuranath et al., 2000), Mini-Mental State Examination (MMSE; Folstein et al., 1975), or Montreal Cognitive Assessment (MoCA; Nasreddine et al., 2005). However, more specific screening instruments are also important (Cullen et al., 2007; Larner, 2013a), notably in diseases where deficits in specific cognitive domains are expected. Dysexecutive impairments, in particular, are an early feature of vascular (e.g., McGuiness et al., 2010) and neurodegenerative dementias (e.g., Baudic et al., 2006; Huey et al., 2009; Tartaglia et al., 2012). Screening tools for executive functions (EF) are thus central for evidence-based approaches in these conditions. In this review, we discuss the four currently available screening instruments for EF: Executive Interview (EXIT 25; Royall et al., 1992), Frontal Assessment Battery (FAB; Dubois et al., 2000), INECO Frontal Screening (IFS; Torralva et al., 2009), and FRONTIER Executive Screen (FES; Leslie et al., 2015).

## Executive functions: definition and neuroanatomical substrates

EF is an umbrella term for higher-order cognitive processes that coordinate cognitive, emotional, and motor activity during the execution of new and complex tasks (Jurado and Rosselli, 2007; Goldstein et al., 2014). Although, their pivotal role in cognition is established, a consensual definition is lacking (Goldstein et al., 2014). A theoretical distinction has been made between metacognitive and emotional EF. Metacognitive (Ardila, 2008; Funkiewiez et al., 2012; Otero and Barker, 2014) or cool EF (Miyake et al., 2000; Zelazo and Muller, 2002) include goal-directed, future-oriented skills such as planning, inhibition, flexibility, set-shifting, and working memory, typically assessed in relatively decontextualized, non-emotional testing conditions. Emotional (Ardila, 2008) or hot (Zelazo and Muller, 2002) EF, in turn, operate in contexts that involve emotion, motivation, or reward-based decision making. However, this distinction might not be straightforward and it has been suggested that metacognitive and emotional EF are closely related and interdependent (Panksepp, 2003; Peterson and Welsh, 2014). According to Panksepp (1998, 2003), they tend to go together in the most of human experiences, with affective/emotional processes providing intrinsic value for the cognitive and behavioral systems (see also Pessoa, 2009).

Neuroimaging studies with healthy adults (e.g., Collette et al., 2006; Burzynska et al., 2012) and brain-damaged patients (e.g., Robinson et al., 2014) indicate that EF are supported by distributed networks including frontal and posterior (mainly parietal) cortical and subcortical regions. For instance, studies using structural techniques found positive correlations between prefrontal cortex (PFC) volume and performance on tasks of EF such as the Wisconsin Card Sorting Test (WCST; Gunning-Dixon and Raz, 2003; Burzynska et al., 2012). Evidence from functional studies (fMRI) implicates the dorsolateral PFC (dlPFC) and anterior cingulate areas in the performance of metacognitive tasks such as the Tower of Hanoi (Unterrainer et al., 2004), digit span (Yoon et al., 2007) and WCST (Wilmsmeier et al., 2010). On the other hand, fMRI studies with patients with frontotemporal degeneration implicate the ventromedial PFC in the mechanisms of emotional/social decision (Grossman et al., 2010; for a review of the PFC involvement in social EF, see Bicks et al., 2015).

Deficits in EF are a common symptom of traumatic (Caeyenberghs et al., 2014), vascular (McGuiness et al., 2010), neuropsychiatric (Reichenberg et al., 2009; Fiorentino et al., 2013; Baez et al., 2014; Caixeta et al., 2017), and neurodegenerative conditions. Concerning neurodegenerative conditions, executive dysfunction is a core symptom of behavioral-variant frontotemporal dementia (bvFTD; Hodges and Miller, 2001; Slachevsky et al., 2004; Fiorentino et al., 2013), and it is also frequent in Parkinson's (Lima et al., 2008; Dirnberger and Jahanshahi, 2013) and Huntington's diseases (You et al., 2014). Metacognitive EF, namely planning, working memory and fluency, seem to be the most affected ones in Parkinson's and Huntington's diseases (Elliott, 2006; Leh et al., 2010; You et al., 2014). On the other hand, bvFTD causes both metacognitive (Huey et al., 2009) and motivational/emotional impairments (Eslinger et al., 2012). Early stage AD patients also show executive dysfunction (e.g., Amiéva et al., 2004), possibly before global cognition deficits are detectable using screening measures such as MMSE (Sgaramella et al., 2001; Stokholm et al., 2006; Clark et al., 2012). Importantly, in these patients, the magnitude of EF dysfunction predicts worse performance in daily living activities, greater need of care, and higher frequency of neuropsychiatric symptoms (Tekin et al., 2001; Swanberg et al., 2004; Stokholm et al., 2006).

Efforts have been made to develop tools to assess executive dysfunction. Although, detailed tests and comprehensive batteries are available (e.g., Behavioral Assessment of Dysexecutive Syndrome, Wilson et al., 1996), the interest in screening tools is relatively more recent. For example, they are not yet discussed in widely used neuropsychological assessment handbooks (e.g., Strauss et al., 2006; Lezak et al., 2012). As compared to comprehensive batteries, screening tools can provide an easier, reliable, and quicker measure of EF, useful in initial assessments, or when the available time and resources are limited.

## Screening tools of executive functions

Four executive screening tools have been devised so far: EXIT 25, FAB, IFS, and FES (see Table 1 for details).

**Table 1:** We reviewed studies focussing on neuropsychological screening tools of executive functions.

	**Executive Interview-25 (EXIT 25)**	**Frontal Assessment Battery (FAB)**	**INECO Frontal Screening (IFS)**	**FRONTIER Executive Screen (FES)**
Original study	Royall et al., 1992	Dubois et al., 2000	Torralva et al., 2009	Leslie et al., 2015
Validation studies for other countries^*^	Mujic et al. (2014, United Kingdom) Azcurra (2013, Argentina) Matioli et al. (2008, Brazil) Chan et al. (2006, China) Sinoff et al. (2001, Israel)	Asaadi et al. (2016, Iran) Benke et al. (2013, German) Beato et al. (2012, Brazil) Chong et al. (2010, China) Kim et al. (2010, Korea) Rodrigues et al. (2009, Brazil) Lima et al. (2008, Portugal) Tunçay et al. (2008, Turkey) Kugo et al. (2007, Japan) Nakaaki et al. (2007, Japan) Appollonio et al. (2005, Italy) Iavarone et al. (2004, Italy) Mok et al. (2004, China)	Custodio et al. (2016, Peru) Moreira et al. (2014, Portugal) Ihnen et al. (2013, Chile)	NA
Normative data	NA	Asaadi et al. (2016, Iran) Beato et al. (2012, Brazil) Kim et al. (2010, Korea) Rodrigues et al. (2009, Brazil) Lima et al. (2008, Portugal) Tunçay et al. (2008, Turkey) Appollonio et al. (2005, Italy) Iavarone et al. (2004, Italy)	Moreira et al. (2014, Portugal)	NA
Cut-offs	NA	12/13 (92% sensitivity and 78% specificity) between healthy elderly and patients with early cognitive impairment (Chong et al., 2010) 11 (76% sensitivity and 79% specificity) between healthy elderly and Huntington's disease patients (Rodrigues et al., 2009) 12 (77% sensitivity and 87% specificity) between AD and FTD patients (Slachevsky et al., 2004)	23.5 (97% sensitivity and 98% specificity) for the detection of dementia and 17.5 (94% sensitivity and 94% specificity) between AD and bvFTD (Custodio et al., 2016) 17 (76% sensitivity and 81% specificity) between healthy elderly and AD patients (Moreira et al., 2014) 25 (96% sensitivity and 92% specificity) between healthy elderly and demented participants and 19 (72% sensitivity and 81% specificity) between bvFTD vs. AD patients (Torralva et al., 2009)	7 (71% sensitivity and 73% specificity) between AD and bvFTD patients (Leslie et al., 2015)
Internal consistency (Chronbach's Alpha)	α = 0.64 (Jahn et al., 2015) α = 0.66 (Campbell et al., 2014) α = 0.72 (Mujic et al., 2014) α = 0.87 (Azcurra (2013) α = 0.86 (Larson et al., 2008) α = 0.80 (Chan et al., 2006) α = 0.87 (Royall et al., 1992)	α = 0.68 (Asaadi et al., 2016) α = 0.69 (Lima et al., 2008) α = 0.78 (Iavarone et al., 2004) α = 0.77 (Mok et al., 2004) α = 0.78 (Dubois et al., 2000)	α = 0.69 (Moreira et al., 2014) α = 0.90 (Ihnen et al., 2013) α = 0.80 (Torralva et al., 2009)	NA
Inter-rater reliability	*r* = 0.98 (Campbell et al., 2014) *r* = 0.96 (Mujic et al., 2014) *r* = 0.94 (Azcurra, 2013) *r* = 0. 91 (Chan et al., 2006) *r* = 0.90 (Royall et al., 1992)	*r* = 0.90 (Asaadi et al., 2016) *r* = 0.96 (Appollonio et al., 2005) *r* = 0.79 (Iavarone et al., 2004) *r* = 0.85 (Mok et al., 2004) *r* = 0.79 (Slachevsky et al., 2004) *r* = 0.87 (Dubois et al., 2000)	*r* = 0.87 (Torralva et al., 2009)	NA
Concurrent validity	*Categorical word fluency* Azcurra (2013): *r* = 0.69 Matioli et al. (2008): *r* = −0.37 Stokholm et al. (2006): *r* = −0.43 *Clock Drawing Test* Campbell et al. (2014): *r* = −0.39 Moorhouse et al. (2009): *r* = −0.61 Matioli et al. (2008): *r* = −0.27 *FAB* Moorhouse et al. (2009): *r* = −0.79 *Lexical word fluency* Azcurra (2013): *r* = 0.67 Stokholm et al. (2006): *r* = −0.54 *MSCT - Categories achieved* Chan et al. (2006): *rho* = −0.54 *MSCT - Perseverative errors* Chan et al. (2006): *r* = 0.29 *Stroop Test* Campbell et al. (2014): *r* = 0.32 Azcurra (2013): *r* = 0.71 Stokholm et al. (2006): *r* = 0.40 *Test of Sustained Attention and Tracking* (time and errors) Royall et al. (1992): *r* = 0.82 & *r* = 0.83, respectively. *TMT A & B* Larson and Heinemann (2010): *r* = 0.44 & *r* = 0.45, respectively Royall et al. (1992): *r* = 0.73 & *r* = 0.64, respectively *TMT-B (time to complete)* Azcurra (2013): *r* = 0.64 *WCST* Campbell et al. (2014): *r* = 0.34 Azcurra (2013): *r* = 0.68 Royall et al. (1992): *r* = 0.52	*Clock Drawing Test* Moorhouse et al. (2009): *r* = 0.59 *DSS* Iavarone et al. (2004): *r* = 0.65 *EXIT−25* Moorhouse et al. (2009): *r* = −0.79 *IFS* Moreira et al. (2014): *r* = 0.92 Ihnen et al. (2013): *r* = 0.94 Gleichgerrcht et al. (2011): *r* = 0.55 *Lexical word fluency* Barulli et al. (2015): *r* = 0.59 Cohen et al. (2012): *r* = 0.66 Rodrigues et al. (2009): *r* = 0.79 Lima et al. (2008): *r* = 0.41 *Mattis DRS* Dubois et al. (2000): *r* = 0.82 *Stroop Test (Interference)* Asaadi et al. (2016): *r* = −0.39 Barulli et al. (2015): *r* = −0.43 Rodrigues et al. (2009): *r* = 0.72 Tunçay et al. (2008): *r* = −0.42 *TMT A & B (time to complete)* Cohen et al. (2012): *r* = −0.66 and *r* = −0.73, respectively Lima et al. (2008): *r* = −0.41 and *r* = −0.41, respectively *TMT-B (time to complete)* Iavarone et al. (2004): *r* = −0.62 *WCST - Perseverative errors* Asaadi et al. (2016): *r* = −0.41 Lima et al. (2008): *r* = −0.43 Mok et al. (2004): *r* = −0.37 Dubois et al. (2000): *rho* = 0.68 *WCST - Number of criteria* Asaadi et al. (2016): *r* = −0.33 Mok et al. (2004): *r* = 0.45 Dubois et al. (2000): *r* = 0.77	*BADS* Zoo Map Test 2 – raw score and execution time Moreira et al. (2014): *r* = 0.43 & *r* = −0.57, respectively Rule Shift Card Errors 1 & 2 Moreira et al. (2014): *r* = −0.39 & *r* = −0.63, respectively *Categorical Word Fluency* Bruno et al. (2015): *r* = 0.45 Ihnen et al. (2013): *r* = 0.73 *Clock Drawing Test* Moreira et al. (2014): *r* = 0.75 *FAB* Moreira et al. (2014): *r* = 0.92 Ihnen et al. (2013): *r* = 0.94 Gleichgerrcht et al. (2011): *r* = 0.55 *MSCT - Categories achieved* Ihnen et al. (2013): *r* = 0.79 *MSCT - Perseverative errors* Ihnen et al. (2013): *r* = −0.62 *Phonological Fluency* Ihnen et al. (2013): A (*r* = 0.68); P (*r* = 0.78) Gleichgerrcht et al. (2011): *r* = 0.63 Torralva et al. (2009): *r* = 0.67 *TMT-B (time to complete)* Bruno et al. (2015): *r* = −0.68 Torralva et al. (2009): *r* = −0.75 Gleichgerrcht et al. (2011): *r* = −0.61 *WCST- Number of categories* Bruno et al. (2015): *r* = 0.48 Torralva et al. (2009): *r* = 0.77 Gleichgerrcht et al. (2011): *r* = 0.73 *WCST - Perseverative errors* Torralva et al. (2009): *r* = −0.77 Gleichgerrcht et al. (2011): *r* = −0.69	*TMT-B* Leslie et al. (2015): *r* = −0.50, *Digit Span Backward* Leslie et al. (2015): *r* = 0.66) Hayling Test Leslie et al. (2015): *r* = 0.73.
Correlations with non-executive measures	*MMSE* Campbell et al. (2014): *r* = −0.44 Mujic et al. (2014): *r* = −0.68 Moorhouse et al. (2009): *r* = 0.61 Matioli et al. (2008): *r* = −0.38 Stokholm et al. (2006): *r* = −0.34 Royall et al. (1992): *r* = −0.85 *MEC* Azcurra (2013): *r* = 0.59 *California Verbal Learning Test* Campbell et al. (2014): *r* = −0.36 *Finger Tapping Test* Campbell et al. (2014): *r* = −0.34.	*ACE-R* Gleichgerrcht et al. (2011): *r* = 0.71 *MMSE* Asaadi et al. (2016): *r* = 0.63 Cohen et al. (2012): *r* = 0.54 Moorhouse et al. (2009): *r* = −0.58 Rodrigues et al. (2009): *r* = 0.83 Lima et al. (2008): *r* = 0.50 Tunçay et al. (2008): *r* = 0.50 Appollonio et al. (2005): *r* = 0.41 Lipton et al. (2005) *r* = 0.53 Mok et al. (2004): *r* = 0.77	*ACE-R* Ihnen et al. (2013): *r* = 0.90 Gleichgerrcht et al. (2011): *r* = 0.42 Torralva et al. (2009): *r* = 0.55 *MMSE* Moreira et al. (2014): *r* = 0.73 Ihnen et al. (2013): *r* = 0.87 Torralva et al. (2009): *r* = 0.57	NA
Correlations with functional and behavioral measures	*Brief Psychiatric Rating Scale* Azcurra (2013): *r* = 0.61 *Frontal Systems Behavior Scale* Azcurra (2013): *r* = 0.62 *Rapid Disability Rating Scale-2* Azcurra (2013): *r* = 0.51 *Cognitive Functional Independence Measure* Larson et al. (2008): *r* = −0.73 *Direct Assessment of Functional Status test* Pereira et al. (2008): *r* = −0.87 *Instrumental Activities of Daily Living* Moorhouse et al. (2009): *r* = 0.40 *Frontal Behavioral Inventory* Stokholm et al. (2006): *r* = 0.38 *Nursing Behaviour Problem Scale* Royall et al. (1992): *r* = 0.79	*Functional Capacity Scale* Rodrigues et al. (2009): *r* = 0.79 *Instrumental Activities of Daily Living* Moorhouse et al. (2009): *r* = −0.46	*Instrumental Activities of Daily Living* Ihnen et al. (2013): *r* = 0.72 *Technologies Activities of Daily Living Questionnaire* Ihnen et al. (2013): *r* = −0.75	NA
Information about neural correlates	Royall et al., 2001	Brugger et al., 2015 Lee et al., 2015 Pellechia et al., 2015 Piatella et al., 2015 Kopp et al., 2013 Oshima et al., 2012 Nagata et al., 2011 Kume et al., 2011 Yoshida et al., 2009 Guedj et al., 2008 Matsui et al., 2006	Baez et al., 2017	NA
Studies with clinical groups^**^	*Alzheimer's disease* Jahn et al., 2015 Azcurra, 2013 Pereira et al., 2008 Stokholm et al., 2006 Royall et al., 2001 Royall et al., 1994 *Dementia (without etiology specification)* Mujic et al., 2014 *Frontotemporal dementia* Azcurra, 2013 Stokholm et al., 2006 Royall et al., 1994 *Mild Cognitive Impairment* Jahn et al., 2015 Pereira et al., 2008 *Mixed dementia* Jahn et al., 2015 Azcurra, 2013 Stokholm et al., 2006 *Traumatic brain injury* Larson et al., 2008 Larson and Heinemann, 2010 *Unipolar major depression* Mujic et al., 2014 Campbell et al., 2014 Royall et al., 1994 *Vascular diseases* Azcurra, 2013 Stokholm et al., 2006 Royall et al., 2001	*Alzheimer's disease* Lee et al., 2015 Boban et al., 2012 Nagata et al., 2011 Yoshida et al., 2009 Tunçay et al., 2008 Oguro et al., 2006 Lipton et al., 2005 Iavarone et al., 2004 Slachevsky et al., 2004 *Amyotrophic lateral sclerosis* Barulli et al., 2015 Ahn et al., 2011 Oskarsson et al., 2010 *Corticobasal degeneration* Dubois et al., 2000 *Dementia with Lewy Bodies* Yoshida et al., 2009 *Frontotemporal dementia* Boban et al., 2012 Yoshida et al., 2009 Guedj et al., 2008 Lipton et al., 2005 Iavarone et al., 2004 Slachevsky et al., 2004 Dubois et al., 2000 *Huntington's disease* Rodrigues et al., 2009 *Mild Cognitive Impairment* Chong et al., 2010 Kume et al., 2011 Yoshida et al., 2009 *Parkinson's disease* Asaadi et al., 2016 Pellechia et al., 2015 Cohen et al., 2012 Marconi et al., 2011 Kenangil et al., 2010 Lima et al., 2008 Tunçay et al., 2008 Matsui et al., 2006 Dubois et al., 2000 *Progressive supranucelar palsy* Piatella et al., 2015 Paviour et al., 2005 Dubois et al., 2000 *Vascular diseases* Kopp et al., 2013 Boban et al., 2012 Yoshida et al., 2009 Oguro et al., 2006 Mok et al., 2004	*Alzheimer's disease* Moreira et al., 2014 Ihnen et al., 2013 Gleichgerrcht et al., 2011 Torralva et al., 2009 *Bipolar disorder* Baez et al., 2017 *Dementia with Lewy Bodies* Ihnen et al., 2013 *Frontotemporal dementia* Baez et al., 2017 Fiorentino et al., 2013 Ihnen et al., 2013 Gleichgerrcht et al., 2011 Torralva et al., 2009 *Vascular diseases* Ihnen et al., 2013 *Mixed dementia* Ihnen et al., 2013 *Major depression* Fiorentino et al., 2013 *Relapsing-Remitting Multiple Sclerosis* Bruno et al., 2015	*Alzheimer's disease* Leslie et al., 2015 *Frontotemporal dementia* Leslie et al., 2015

*The selection was based on the authors' knowledge of the literature, and on an extensive search in the PubMed database (www.pubmed.com), using strings such as “executive functions screening tools,” “executive screening tools,” and “brief assessment of executive functions.” We also conducted searches using the names of the screening tools, after they were identified in a first search, namely “Executive Interview—25,” “EXIT-25,” Frontal Assessment Battery,” “FAB,” “INECO Frontal Screening,” “IFS,” “FRONTIER Executive Screen,” and “FES.” We only included studies that were published in English and that examined samples of elderly participants with or without neurologic or psychiatric disorders. Studies related to The Frontal Lobe Score (Ettlin et al., 2000) were not included because this tool takes 30–60 min to administer, and this largely exceeds the recommended duration of screening tools (<15 min; Malloy et al., 1997). We also did not include studies related to the Clock Drawing Test, as this test is not consistently considered as a measure of EF in the literature (Lezak et al., 2012). ACE-R, Addenbrooke Cognitive Examination- Revised; AD, Alzheimer's Disease; BADS, Behavioral Assessment of Dysexecutive Syndrome; DRS, Dementia Rating Scale; DSS, Digit-Symbol Substitution; FTD, Frontotemporal Dementia; MEC, Mini-Examen Cognoscitivo; MMSE, Mini Mental State Examination; MSCT, Modified Cards Sorting Test; NA, Non-Available; TMT, Trail Making Test; WCST, Wisconsin Cards Sorting Test. Only significant correlations are reported in the table*.

* *We considered as validation studies papers that described in the methods section the adaptation/validation of the executive screening tool for the population of a specific country*.

** *These papers illustrate the available evidence across different clinical conditions, but the selection is not intended to be an exhaustive and systematic review of the literature*.

EXIT25 (Royall et al., 1992) was the first screening tool designed to assess EF, including working memory, verbal and visual fluency, inhibitory control, motor programming, and imitation behavior. It consists of 25 items and takes around 15 min to administer. The scores range from 0 to 50, with higher values indicating worse performance. The validation study compared EXIT 25 scores across elderly groups with different levels of functional dependency. Contrary to the MMSE, EXIT 25 discriminated between groups, and presented good psychometric properties, namely good internal consistency, inter-rater reliability, and strong correlations with standard EF measures, including the Trail Making Test B (TMT-B), the Test of Sustained Attention, and the WCST (Ray et al., 1992; Royall et al., 1992). Associations between higher EXIT 25 scores and disruptive behaviors (Ray et al., 1992), functional decline (Royall et al., 2004; Pereira et al., 2008), and probability of dementia (Stokholm et al., 2006) were also found. Left anterior frontal lobes lesions related to worse EXIT 25 performance in studies with vascular and AD patients, even when controlling for age, dementia type and severity (Royall et al., 2001). Further studies also confirmed EXIT 25's ability to detect executive dysfunction in neurodegenerative (e.g., AD, FTD; Stokholm et al., 2006; Azcurra, 2013) and non-degenerative (e.g., traumatic brain injury; Larson et al., 2008) conditions. However, it failed to differentiate between patients with probable AD with and without major depression, vascular dementia without cortical features, and schizophrenia (Royall et al., 1994). Another limitation of EXIT 25 is its significant correlations with non-EF measures such as the MMSE (Royall et al., 1992; Matioli et al., 2008; Campbell et al., 2014), the California Verbal Learning Test and the Finger Taping Test (Campbell et al., 2014). This could indicate poor specificity (Dubois et al., 2000). Campbell et al. (2014) argued that although poor scores on EXIT 25 indicate cognitive deficits with an executive component, it is not a specific measure of EF alone. There are validation studies for countries like the United Kingdom (Mujic et al., 2014), Argentina (Azcurra, 2013), Brazil (Matioli et al., 2008), China (Chan et al., 2006), and Israel (Sinoff et al., 2001), but normative data and cut-offs were not provided. Shorter versions have been presented. The Quick EXIT (Larson and Heinemann, 2010) incudes 14-items and shows good internal consistency (Cronbach's alpha = 0.88) and moderate correlations with other measures of EF such as TMT (Campbell et al., 2014). The EXIT 8 (Jahn et al., 2015) is an 8-item version that shows good internal consistency (Cronbach's alpha = 0.74), high correlation with the full EXIT 25, and good ability to discriminate controls from patients (Area Under Curve—AUC = 0.81 for Mild Cognitive Impairment, and AUC = 0.92 for dementia). EXIT 8 remains a significant predictor of other EF measures after controlling for MMSE scores, which suggests that it might be a robust measure. Despite the good indicators, studies considering other clinical populations are warranted.

To derive a more specific tool than EXIT 25, Dubois et al. (2000) developed the FAB, which became the most widely used screening tool for EF. It takes around 10 min to administer, and consists of six subtests that assess conceptualization, mental flexibility, motor programming, sensitivity to interference, inhibitory control, and environmental autonomy. Higher values (minimum of 0 and maximum of 18) indicate better EF. The FAB presented good inter-rater reliability and internal consistency, strong positive correlations with the WCST, and a good discriminative ability between controls and patients with Parkinson's disease, corticobasal degeneration, FTD, and progressive supranuclear palsy (Dubois et al., 2000). Its psychometric properties were further inspected: strong correlations were found with measures such as TMT, verbal fluency (Lima et al., 2008; Rodrigues et al., 2009) and Stroop Test (Rodrigues et al., 2009), as well as high inter-rater reliability (Appollonio et al., 2005), and high accuracy in differentiating controls from patients with FTD (Slachevsky et al., 2004; Lipton et al., 2005), Parkinson's disease (PD; Lima et al., 2008), AD (Slachevsky et al., 2004; Guedj et al., 2008), Huntington's disease (Rodrigues et al., 2009), and amyotrophic lateral sclerosis (Barulli et al., 2015). Associations were found between FAB performance and regional cerebral glucose metabolism in dlPFC and middle frontal gyri areas in AD patients (Oshima et al., 2012; Lee et al., 2015), with dorsolateral prefrontal cortex and parietal lobe areas in PD patients (Brugger et al., 2015), and with perfusion in the dlPFC, medial premotor cortex and anterior cingulate cortex in bvFTD patients (Guedj et al., 2008; for a review of FAB neural correlates see Hurtado-Pomares et al., 2017). Different versions of this tool and normative data are available, for example, in Iranian (Asaadi et al., 2016), German (Benke et al., 2013), Brazilian Portuguese (Beato et al., 2012), Korean (Kim et al., 2010), European Portuguese (Lima et al., 2008), Turkish (Tunçay et al., 2008), Japanese (Kugo et al., 2007), Italian (Iavarone et al., 2004; Appollonio et al., 2005), and Chinese (Mok et al., 2004; Chong et al., 2010). A cut-off of 12 distinguished between FTD and AD patients with good sensitivity and specificity (Iavarone et al., 2004; Slachevsky et al., 2004). For patients with Huntington's disease, a cut-off of 10/11 achieved the best sensitivity and specificity (Rodrigues et al., 2009). In comparisons with EXIT 25, it was noticed that despite their similarities, FAB was briefer and easier to administer in a memory clinic setting (Moorhouse et al., 2009). Its contribution for differential diagnosis remains non-consensual, however. FAB differentiated patients with AD and vascular dementia (Oguro et al., 2006). Concerning byFTD and AD patients, although bvFTD presented lower scores in some studies (Iavarone et al., 2004; Slachevsky et al., 2004; Nakaaki et al., 2007), in other studies differences were not significant (Castiglioni et al., 2006; Gleichgerrcht et al., 2011). Additionally, some of its subtests have been shown to have poor sensitivity (e.g., Prehension Behaviour and Letter Fluency; Lima et al., 2008; Moreira et al., 2014). Age (Iavarone et al., 2004; Appollonio et al., 2005; Lima et al., 2008) and education (Iavarone et al., 2004; Appollonio et al., 2005; Rodrigues et al., 2009) should be considered when interpreting FAB scores, since they are predictors of performance, with advanced age and less years of education predicting lower scores. FAB correlates with MMSE in many studies (e.g., Lima et al., 2008; Moorhouse et al., 2009; Rodrigues et al., 2009), suggesting that, like EXIT 25, this measure relates to other cognitive domains.

More recently, Torralva et al. (2009) developed the Institute of Cognitive Neurology (INECO) Frontal Screening (IFS). The IFS takes around 10 min to administer, includes eight subtests that cover three executive domains (response inhibition and set shifting, abstraction, and working memory), and assess processes such as motor programming, sensitivity to interference, inhibitory control, verbal inhibitory control, abstraction, and working memory. Three subtests (Motor series, Conflicting instructions, and Go-no-go) were taken from FAB, whereas the remaining ones (Backward digit span; Months of the year backward; Modified Corsi block, Proverb interpretation and Modified Hayling test) were selected to optimize sensitivity. The total score ranges from 0 to 30, with higher scores indicating better performance. Psychometric properties were reported: good internal consistency and concurrent validity, as shown by strong correlations with standard measures of EF such as the WCST, TMT-B (Torralva et al., 2009; Gleichgerrcht et al., 2011) and Lexical and Semantic Fluency (Torralva et al., 2009; Ihnen et al., 2013). Additionally, associations with functional measures have been found, such as the Activities of Daily Living Scale (Ihnen et al., 2013). The IFS discriminate between healthy controls and patients with bvFTD (Torralva et al., 2009; Gleichgerrcht et al., 2011; Fiorentino et al., 2013; Custodio et al., 2016), AD (Torralva et al., 2009; Gleichgerrcht et al., 2011; Moreira et al., 2014; Custodio et al., 2016), relapsing–remitting multiple sclerosis (Bruno et al., 2015), bipolar disorder and ADHD (Baez et al., 2014), as well as between clinical conditions, with bvFTD patients scoring lower than those with major depression (Fiorentino et al., 2013) and AD (Torralva et al., 2009; Custodio et al., 2016). Cut-offs have been presented throughout the studies, with overall values of sensitivity and specificity above 70%: 23.5 (Custodio et al., 2016) and 25 (Torralva et al., 2009) for the detection of dementia, and 17.5 (Custodio et al., 2016) and 19 (Torralva et al., 2009) for the discrimination between bvFTD and AD patients. In a sample with lower education, Moreira et al. (2014) showed that an optimal cut-off of 17 discriminated between healthy controls from AD patients. Only one study explored the neural correlates of IFS, finding that total scores were associated with atrophy in the amygdala, the hippocampus, the parahippocampal gyrus, the fusiform gyrus, and the orbitofrontal cortex in bvFTD patients (Baez et al., 2017). Comparisons between IFS and FAB were undertaken, and these have shown that IFS is more sensitive and specific in differentiating bvFTD from AD (Gleichgerrcht et al., 2011; Custodio et al., 2016), and it also correlates more strongly with standard executive tasks (e.g., TMT-B, WCST, Gleichgerrcht et al., 2011). Nonetheless, the advantage of IFS over FAB remains to be established: in a study with AD patients, they showed similar diagnostic accuracy (0.88 and 0.87, respectively) and similar correlations with other measures of EF (Moreira et al., 2014). IFS scores also correlate with MMSE scores (Torralva et al., 2009; Ihnen et al., 2013; Moreira et al., 2014) and are influenced by age (Moreira et al., 2014) and education (Ihnen et al., 2013; Moreira et al., 2014), reinforcing the need of normative data to take these variables into account. To our knowledge, though, normative data are only available for the Portuguese IFS (Moreira et al., 2014).

Finally, Leslie et al. (2015) developed the FRONTIER Executive Screen (FES), a tool that combines tasks believed to differentiate bvFTD from AD, namely verbal fluency, inhibition, and working memory. The scores range from 0 to 15, with higher values indicating better performance. The FES showed good discriminant accuracy between controls and patients, and between AD and bvFTD groups (a cut-off of 7 reached good sensitivity and specificity). Strong correlations with standard EF measures were found (TMT-B, Digit Span Backward, and the Hayling Test). However, compared with the other screening tools, FES covers less executive domains, and this could reduce its sensitivity. Additionally, the highly specific FES goal—to differentiate patients with bvFTD and AD—, along with the absence of normative data, may limit its clinical usefulness. Studies focused on the influence of sociodemographic variables in FES performance and on its neural correlates remain to be conducted as well.

## Discussion and future directions

Deficits in EF are a symptom of several disorders and screening tools are a promising method for their reliable and fast assessment. We have discussed the four screening tools of EF currently available. A common feature to the discussed tools is the emphasis on metacognitive EF. Thus, for patients with deficits in affective/social components (e.g., bvFTD; Rahman et al., 1999; Eslinger et al., 2012), they might provide more limited information. The Social Cognition and Emotional Assessment (SEA; Funkiewiez et al., 2012) and mini SEA (Bertoux et al., 2012) could be an option in these cases, though they take longer than 30 min to be completed.

Special attention has been given to the psychometric properties of screening tools of EF, as these are critical to determine their clinical utility (Cullen et al., 2007; Larner, 2013a). All the discussed tools show good psychometric properties according to the available guidelines (Malloy et al., 1997). Apart from the FES, that still lacks this analysis, all of them show good internal consistency and inter-rater reliability. The IFS and FES show similar accuracy in detecting executive impairments in bvFTD as compared to AD. In turn, FES seems to be more prone to wrong classifications, as indicated by its lower specificity. Correlations with standard executive measures were consistently found. However, all the executive screening tools also presented correlations with measures of global cognition like the MMSE. This has been pointed out as a limitation, i.e., a sign of low specificity. Nonetheless, considering the multifaceted nature of EF, it is reasonable to expect that they influence performance in global cognitive measures, as these include executive components themselves. More studies will be needed to establish whether screening tools of EF provide information that is useful over and above that provided by global cognitive measures.

The availability of normative data is key for clinical practice: conclusions about deficits are more reliable if performance is compared against population data matched for age and education. Unfortunately, insufficient attention has been given to this. Only FAB and IFS presented normative studies, and only in some of the countries where they have been validated. This limits the utility of these tools and deserves more attention in future work.

Another point that deserves more attention in future research is the relative usefulness of executive screening tools in the different stages of neurodegenerative diseases. Progression generally occurs toward generalized deficits (Horton and Wedding, 2008), and this makes the interpretation (and diagnostic value) of domain-specific assessments more challenging. Executive screening tools could be useful for differential diagnosis in earlier stages of the disease (when combined with other measures), while their contribution in later stages could be more related to the description of the neurocognitive phenotype, i.e., the pattern of relatively preserved and impaired functions.

The relationship between screening tools and brain structure and function remains poorly explored as well. This is crucial to examine whether these tools recruit the same systems that have been identified in experimental cognitive neuroscience research. For EXIT 25 and FAB, there is some evidence of associations with prefrontal structures. However, this remains poorly explored for the IFS and unknown for the FES.

Apart from FES, which is briefer, the remaining executive screening tools are relatively similar concerning structure, time of application, covered domains, and psychometric properties. More comparative research will be critical, across different clinical groups, to establish in which conditions each of the available tools is most useful. Larner (2013b), for example, compared some screening measures of global cognition, providing conclusions that are highly valuable for clinicians. Some attempts to compare FAB, EXIT 25, and IFS in AD and bvFTD patients have been made, but conclusive evidence is still missing.

## Author contributions

HM, AC, CL, and SV contributed to the conception and design of the work. HM prepared the first draft of the work, and AC, SC, CL, and SV revised it critically for important intellectual content. All authors approved the final version of the manuscript.

### Conflict of interest statement

The authors declare that the research was conducted in the absence of any commercial or financial relationships that could be construed as a potential conflict of interest.
